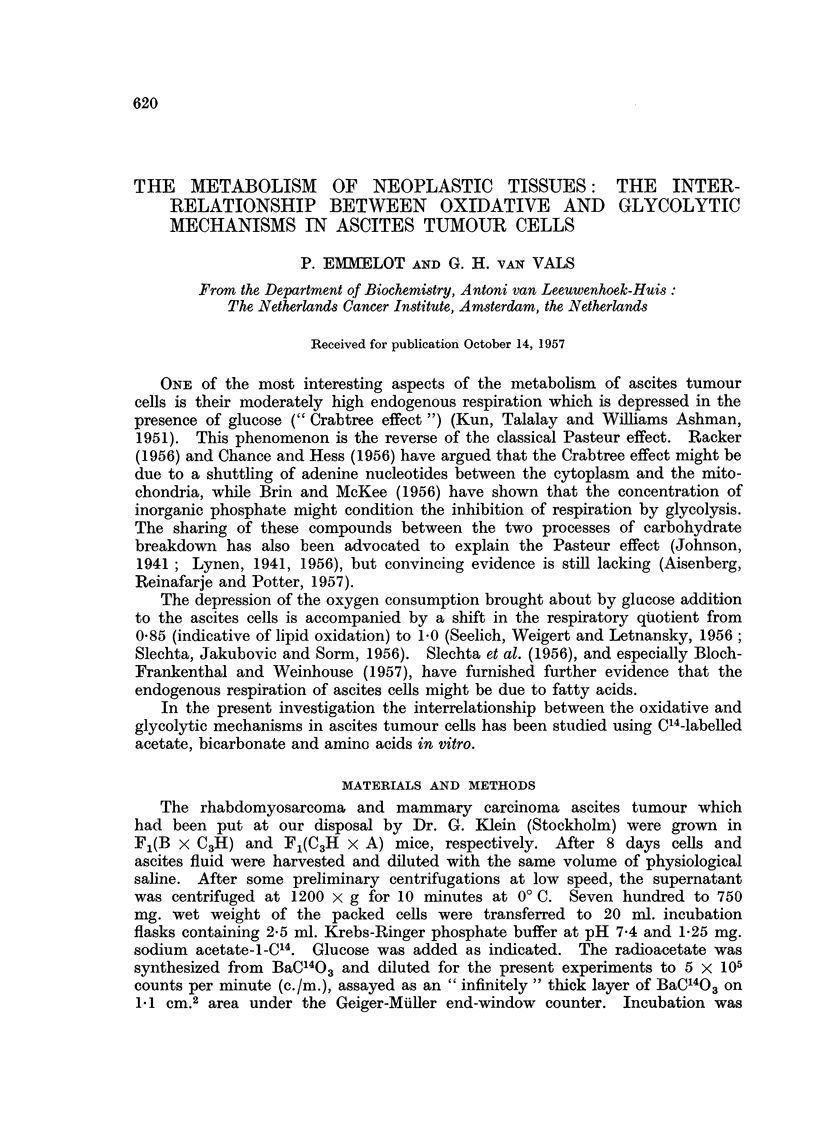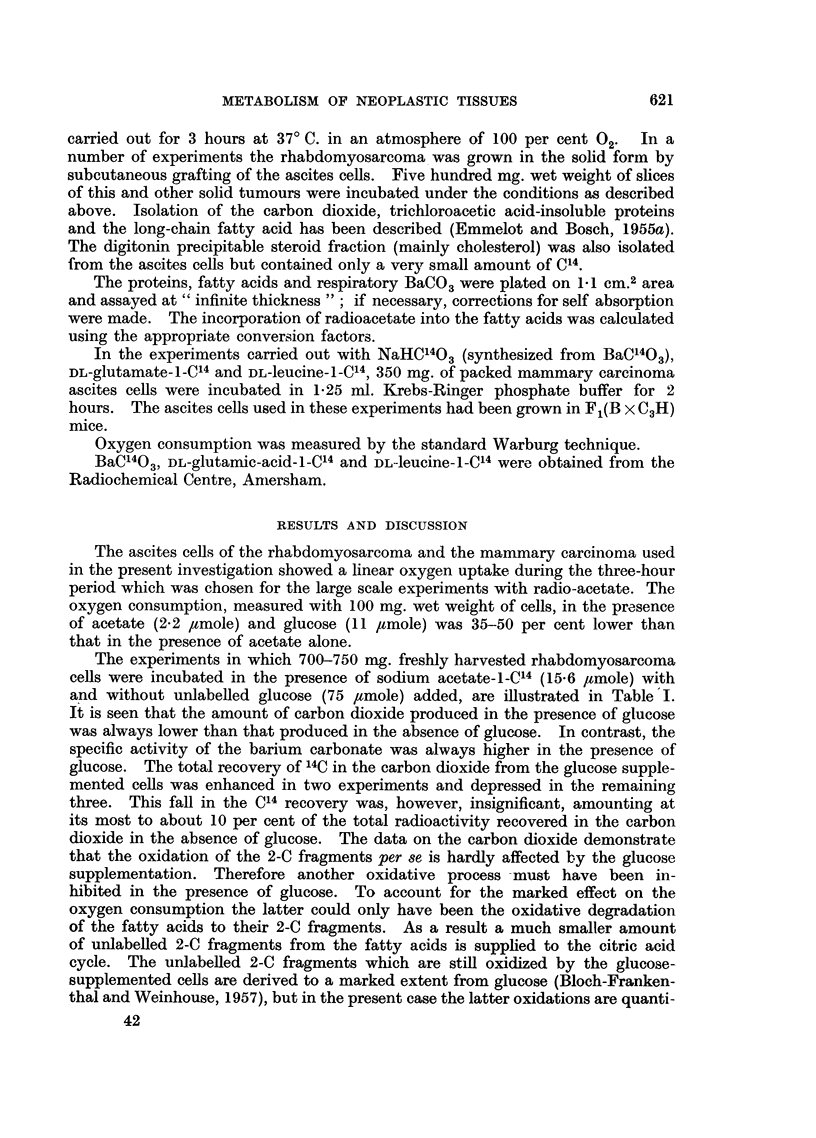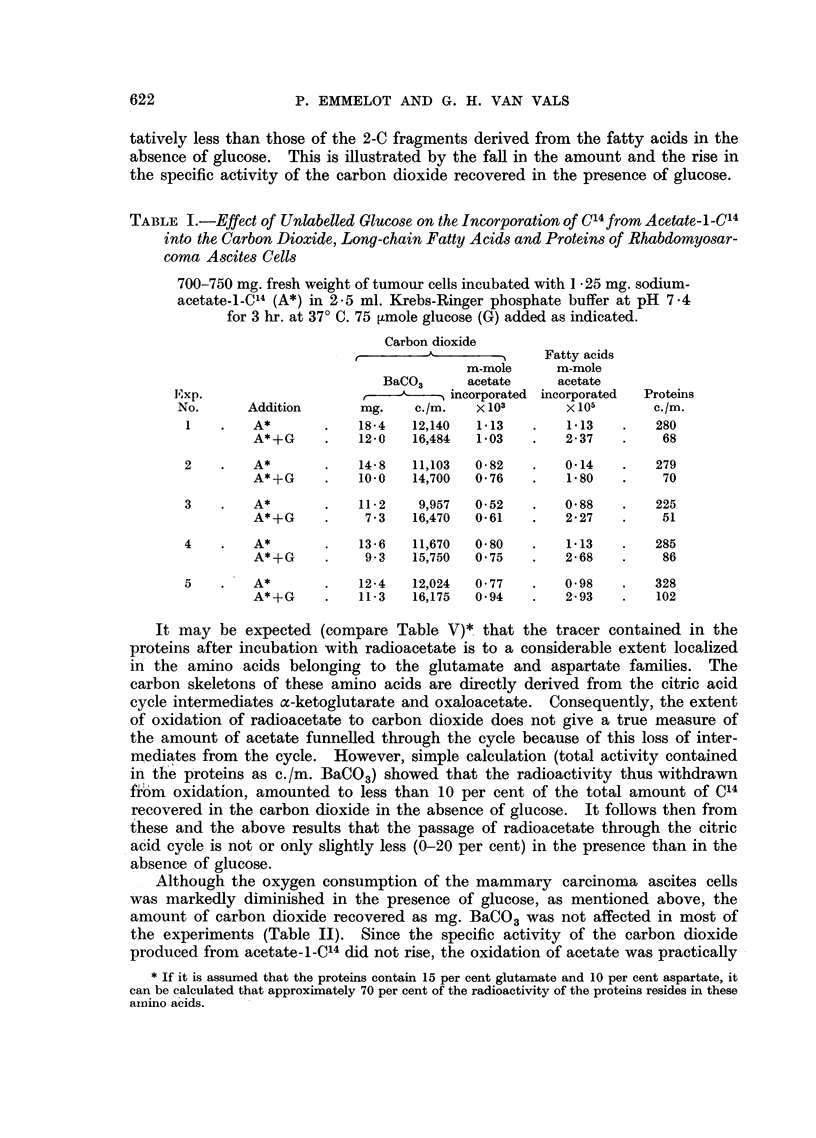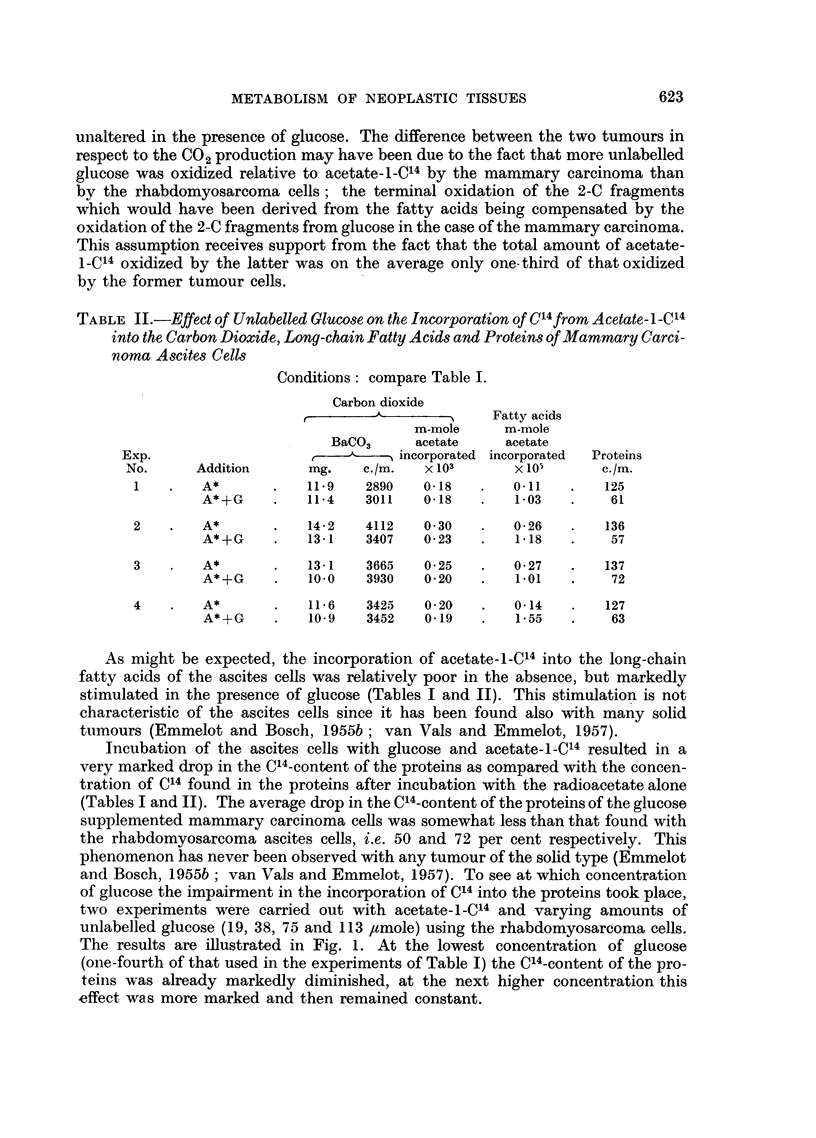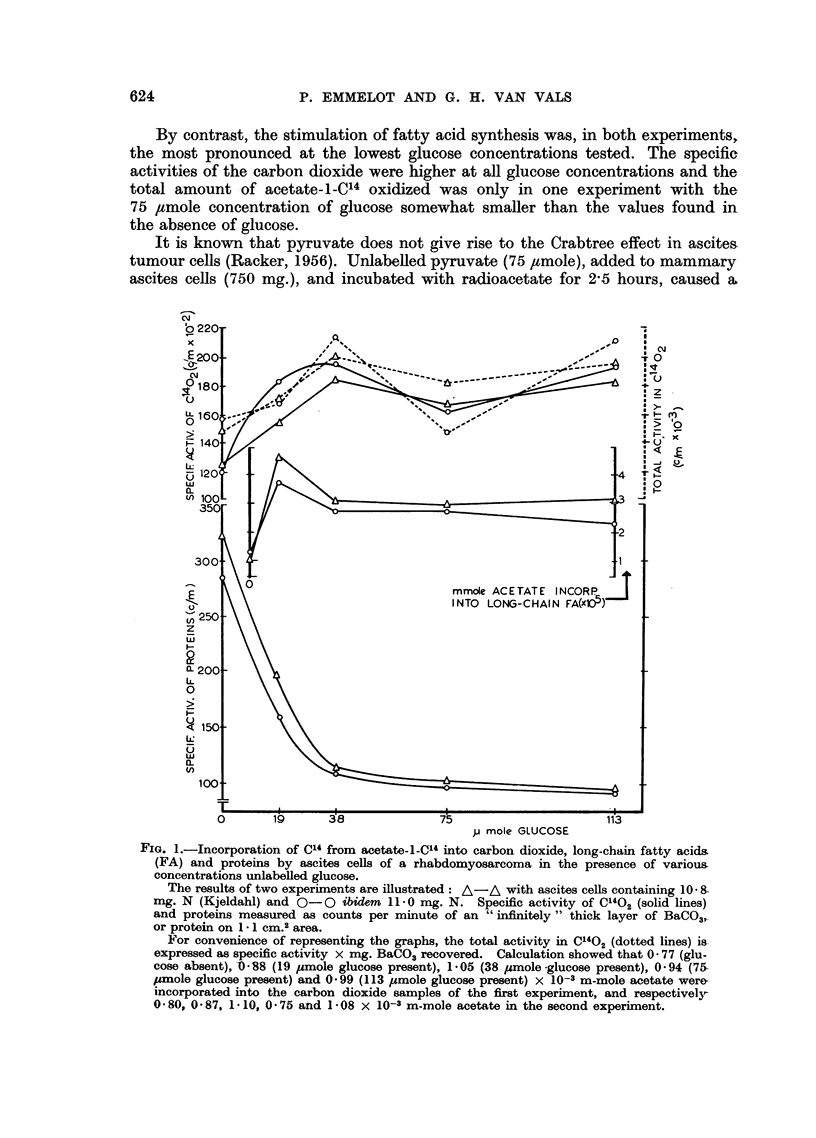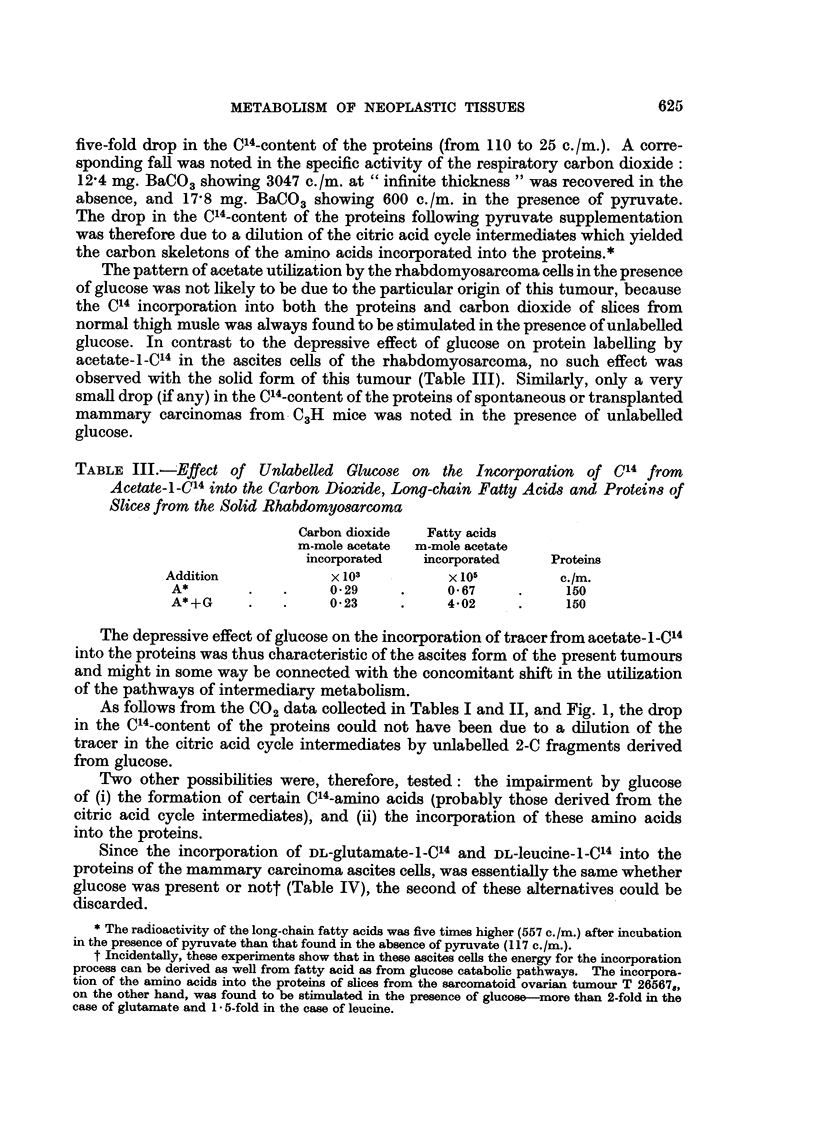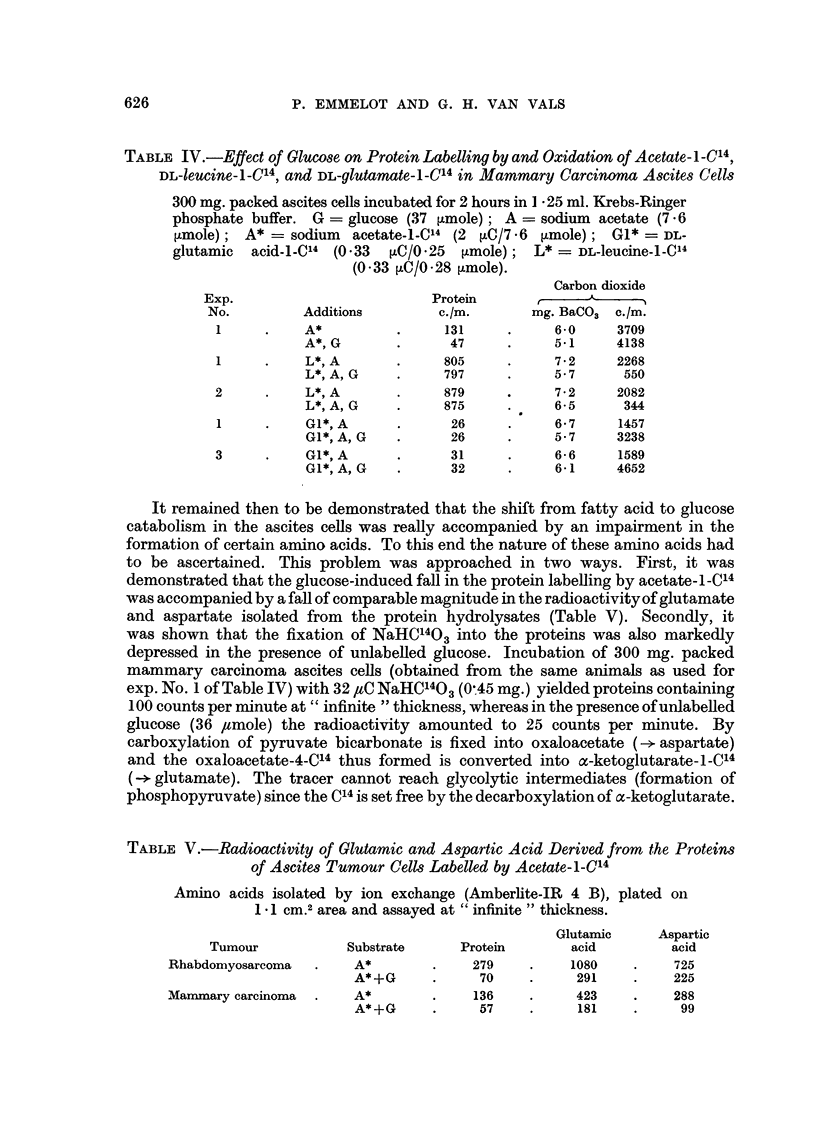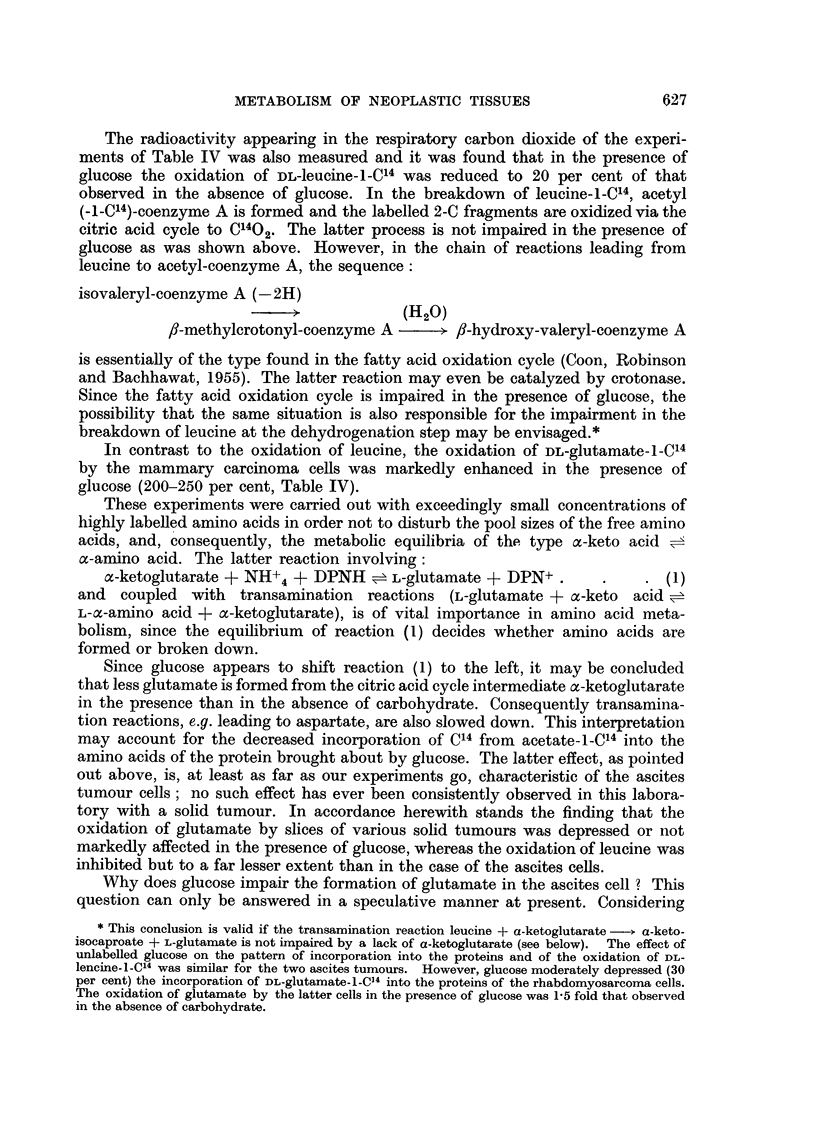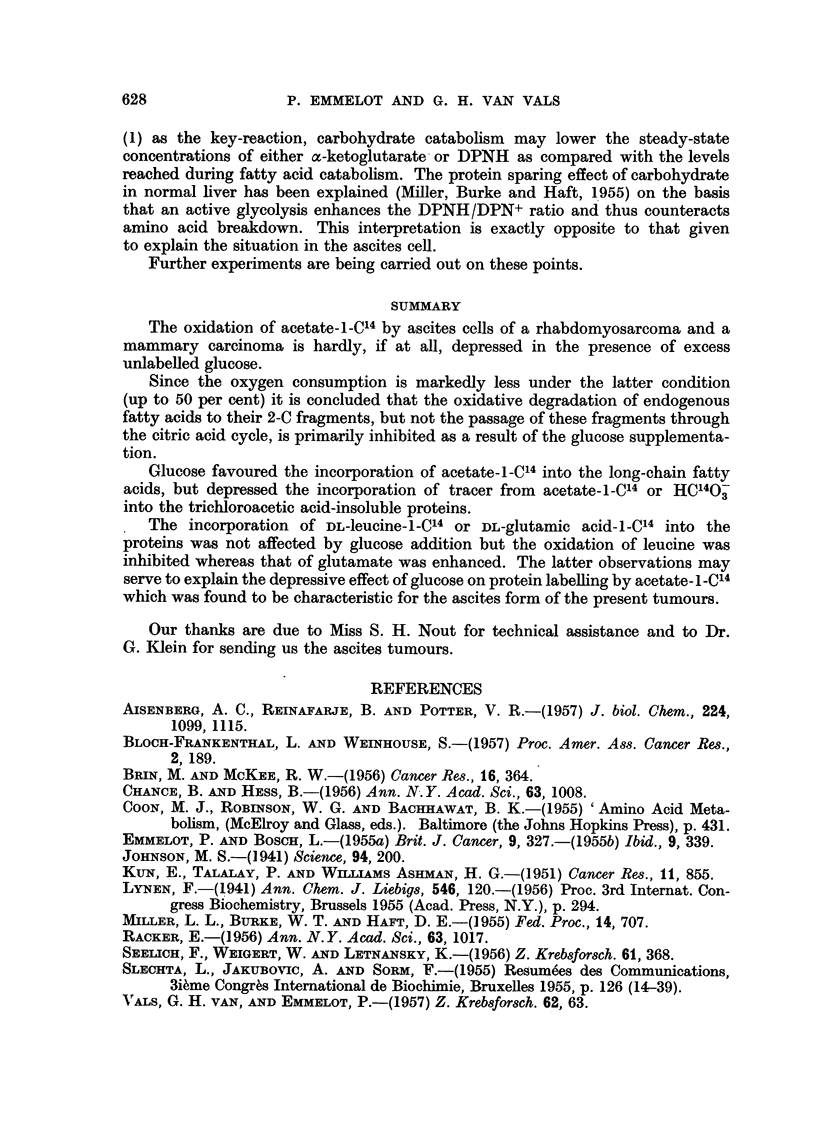# The Metabolism of Neoplastic Tissues: the Inter-relationship between Oxidative and Glycolytic Mechanisms in Ascites Tumour Cells

**DOI:** 10.1038/bjc.1957.76

**Published:** 1957-12

**Authors:** P. Emmelot, G. H. van Vals


### THE METABOLISM OF NEOPLASTIC TISSUES: THE INTERRELATIONSHIP BETWEEN OXIDATIVE AND GLYCOLYTIC MECHANISMS IN ASCITES TUMOUR CELLS

P. EMMELOT AND G. H. VAN VALS

*From the Department of Biochemistry, Antoni van Leeuwenhoek-Huis: The Netherlands Cancer Institute, Amsterdam, the Netherlands*

Received for publication October 14, 1957

ONE of the most interesting aspects of the metabolism of ascites tumour cells is their moderately high endogenous respiration which is depressed in the presence of glucose (" Crabtree effect ") (Kun, Talalay and Williams Ashman, 1951). This phenomenon is the reverse of the classical Pasteur effect. Racker (1956) and Chance and Hess (1956) have argued that the Crabtree effect might be due to a shuttling of adenine nucleotides between the cytoplasm and the mitochondria, while Brin and McKee (1956) have shown that the concentration of inorganic phosphate might condition the inhibition of respiration by glycolysis. The sharing of these compounds between the two processes of carbohydrate breakdown has also been advocated to explain the Pasteur effect (Johnson, 1941; Lynen, 1941, 1956), but convincing evidence is still lacking (Aisenberg, Reinafarje and Potter, 1957).

The depression of the oxygen consumption brought about by glucose addition to the ascites cells is accompanied by a shift in the respiratory quotient from 0·85 (indicative of lipid oxidation) to 1·0 (Seelich, Weigert and Letnansky, 1956; Slechta, Jakubovic and Sorm, 1956). Slechta *et al.* (1956), and especially Bloch-Frankenthal and Weinhouse (1957), have furnished further evidence that the endogenous respiration of ascites cells might be due to fatty acids.

In the present investigation the interrelationship between the oxidative and glycolytic mechanisms in ascites tumour cells has been studied using $C^{14}$-labelled acetate, bicarbonate and amino acids *in vitro.*

### MATERIALS AND METHODS

The rhabdomyosarcoma and mammary carcinoma ascites tumour which had been put at our disposal by Dr. G. Klein (Stockholm) were grown in $F_1(B \times C_3H)$ and $F_1(C_3H \times A)$ mice, respectively. After 8 days cells and ascites fluid were harvested and diluted with the same volume of physiological saline. After some preliminary centrifugations at low speed, the supernatant was centrifuged at $1200 \times g$ for 10 minutes at 0° C. Seven hundred to 750 mg. wet weight of the packed cells were transferred to 20 ml. incubation flasks containing 2·5 ml. Krebs-Ringer phosphate buffer at pH 7·4 and 1·25 mg. sodium acetate-1-$C^{14}$. Glucose was added as indicated. The radioacetate was synthesized from $BaC^{14}O_3$ and diluted for the present experiments to $5 \times 10^5$ counts per minute (c./m.), assayed as an " infinitely " thick layer of $BaC^{14}O_3$ on 1·1 cm.$^2$ area under the Geiger-Müller end-window counter. Incubation was carried out for 3 hours at 37° C. in an atmosphere of 100 per cent $O_2$. In a number of experiments the rhabdomyosarcoma was grown in the solid form by subcutaneous grafting of the ascites cells. Five hundred mg. wet weight of slices of this and other solid tumours were incubated under the conditions as described above. Isolation of the carbon dioxide, trichloroacetic acid-insoluble proteins and the long-chain fatty acid has been described (Emmelot and Bosch, 1955*a*). The digitonin precipitable steroid fraction (mainly cholesterol) was also isolated from the ascites cells but contained only a very small amount of $C^{14}$.

The proteins, fatty acids and respiratory $BaCO_3$ were plated on 1·1 cm.$^2$ area and assayed at " infinite thickness " ; if necessary, corrections for self absorption were made. The incorporation of radioacetate into the fatty acids was calculated using the appropriate conversion factors.

In the experiments carried out with $NaHC^{14}O_3$ (synthesized from $BaC^{14}O_3$), DL-glutamate-1-$C^{14}$ and DL-leucine-1-$C^{14}$, 350 mg. of packed mammary carcinoma ascites cells were incubated in 1·25 ml. Krebs-Ringer phosphate buffer for 2 hours. The ascites cells used in these experiments had been grown in $F_1(B \times C_3H)$ mice.

Oxygen consumption was measured by the standard Warburg technique.

$BaC^{14}O_3$, DL-glutamic-acid-1-$C^{14}$ and DL-leucine-1-$C^{14}$ were obtained from the Radiochemical Centre, Amersham.

### RESULTS AND DISCUSSION

The ascites cells of the rhabdomyosarcoma and the mammary carcinoma used in the present investigation showed a linear oxygen uptake during the three-hour period which was chosen for the large scale experiments with radio-acetate. The oxygen consumption, measured with 100 mg. wet weight of cells, in the presence of acetate (2·2 $\mu$mole) and glucose (11 $\mu$mole) was 35–50 per cent lower than that in the presence of acetate alone.

The experiments in which 700–750 mg. freshly harvested rhabdomyosarcoma cells were incubated in the presence of sodium acetate-1-$C^{14}$ (15·6 $\mu$mole) with and without unlabelled glucose (75 $\mu$mole) added, are illustrated in Table I. It is seen that the amount of carbon dioxide produced in the presence of glucose was always lower than that produced in the absence of glucose. In contrast, the specific activity of the barium carbonate was always higher in the presence of glucose. The total recovery of $^{14}C$ in the carbon dioxide from the glucose supplemented cells was enhanced in two experiments and depressed in the remaining three. This fall in the $C^{14}$ recovery was, however, insignificant, amounting at its most to about 10 per cent of the total radioactivity recovered in the carbon dioxide in the absence of glucose. The data on the carbon dioxide demonstrate that the oxidation of the 2-C fragments *per se* is hardly affected by the glucose supplementation. Therefore another oxidative process must have been inhibited in the presence of glucose. To account for the marked effect on the oxygen consumption the latter could only have been the oxidative degradation of the fatty acids to their 2-C fragments. As a result a much smaller amount of unlabelled 2-C fragments from the fatty acids is supplied to the citric acid cycle. The unlabelled 2-C fragments which are still oxidized by the glucose-supplemented cells are derived to a marked extent from glucose (Bloch-Frankenthal and Weinhouse, 1957), but in the present case the latter oxidations are quantitatively less than those of the 2-C fragments derived from the fatty acids in the absence of glucose. This is illustrated by the fall in the amount and the rise in the specific activity of the carbon dioxide recovered in the presence of glucose.

TABLE I.—*Effect of Unlabelled Glucose on the Incorporation of $C^{14}$ from Acetate-1-$C^{14}$ into the Carbon Dioxide, Long-chain Fatty Acids and Proteins of Rhabdomyosarcoma Ascites Cells*

700–750 mg. fresh weight of tumour cells incubated with 1·25 mg. sodium-acetate-1-$C^{14}$ (A*) in 2·5 ml. Krebs-Ringer phosphate buffer at pH 7·4 for 3 hr. at 37° C. 75 μmole glucose (G) added as indicated.

| | | Carbon dioxide | | | Fatty acids | |
|---|---|---|---|---|---|---|
| | | $BaCO_3$ | | m-mole acetate | m-mole acetate | |
| Exp. No. | Addition | mg. | c./m. | incorporated ×10³ | incorporated ×10⁵ | Proteins c./m. |
| 1 | A* | 18·4 | 12,140 | 1·13 | 1·13 | 280 |
| | A*+G | 12·0 | 16,484 | 1·03 | 2·37 | 68 |
| 2 | A* | 14·8 | 11,103 | 0·82 | 0·14 | 279 |
| | A*+G | 10·0 | 14,700 | 0·76 | 1·80 | 70 |
| 3 | A* | 11·2 | 9,957 | 0·52 | 0·88 | 225 |
| | A*+G | 7·3 | 16,470 | 0·61 | 2·27 | 51 |
| 4 | A* | 13·6 | 11,670 | 0·80 | 1·13 | 285 |
| | A*+G | 9·3 | 15,750 | 0·75 | 2·68 | 86 |
| 5 | A* | 12·4 | 12,024 | 0·77 | 0·98 | 328 |
| | A*+G | 11·3 | 16,175 | 0·94 | 2·93 | 102 |

It may be expected (compare Table V)* that the tracer contained in the proteins after incubation with radioacetate is to a considerable extent localized in the amino acids belonging to the glutamate and aspartate families. The carbon skeletons of these amino acids are directly derived from the citric acid cycle intermediates α-ketoglutarate and oxaloacetate. Consequently, the extent of oxidation of radioacetate to carbon dioxide does not give a true measure of the amount of acetate funnelled through the cycle because of this loss of intermediates from the cycle. However, simple calculation (total activity contained in the proteins as c./m. $BaCO_3$) showed that the radioactivity thus withdrawn from oxidation, amounted to less than 10 per cent of the total amount of $C^{14}$ recovered in the carbon dioxide in the absence of glucose. It follows then from these and the above results that the passage of radioacetate through the citric acid cycle is not or only slightly less (0–20 per cent) in the presence than in the absence of glucose.

Although the oxygen consumption of the mammary carcinoma ascites cells was markedly diminished in the presence of glucose, as mentioned above, the amount of carbon dioxide recovered as mg. $BaCO_3$ was not affected in most of the experiments (Table II). Since the specific activity of the carbon dioxide produced from acetate-1-$C^{14}$ did not rise, the oxidation of acetate was practically

* If it is assumed that the proteins contain 15 per cent glutamate and 10 per cent aspartate, it can be calculated that approximately 70 per cent of the radioactivity of the proteins resides in these amino acids.

unaltered in the presence of glucose. The difference between the two tumours in respect to the $CO_2$ production may have been due to the fact that more unlabelled glucose was oxidized relative to acetate-1-$C^{14}$ by the mammary carcinoma than by the rhabdomyosarcoma cells ; the terminal oxidation of the 2-C fragments which would have been derived from the fatty acids being compensated by the oxidation of the 2-C fragments from glucose in the case of the mammary carcinoma. This assumption receives support from the fact that the total amount of acetate-1-$C^{14}$ oxidized by the latter was on the average only one-third of that oxidized by the former tumour cells.

TABLE II.—*Effect of Unlabelled Glucose on the Incorporation of $C^{14}$ from Acetate-*1-$C^{14}$ *into the Carbon Dioxide, Long-chain Fatty Acids and Proteins of Mammary Carcinoma Ascites Cells*

Conditions : compare Table I.

| Exp. No. | Addition | Carbon dioxide: $BaCO_3$ mg. | Carbon dioxide: $BaCO_3$ c./m. | Carbon dioxide: m-mole acetate incorporated $\times 10^3$ | Fatty acids m-mole acetate incorporated $\times 10^5$ | Proteins c./m. |
|---|---|---|---|---|---|---|
| 1 | A* | 11·9 | 2890 | 0·18 | 0·11 | 125 |
| | A*+G | 11·4 | 3011 | 0·18 | 1·03 | 61 |
| 2 | A* | 14·2 | 4112 | 0·30 | 0·26 | 136 |
| | A*+G | 13·1 | 3407 | 0·23 | 1·18 | 57 |
| 3 | A* | 13·1 | 3665 | 0·25 | 0·27 | 137 |
| | A*+G | 10·0 | 3930 | 0·20 | 1·01 | 72 |
| 4 | A* | 11·6 | 3425 | 0·20 | 0·14 | 127 |
| | A*+G | 10·9 | 3452 | 0·19 | 1·55 | 63 |

As might be expected, the incorporation of acetate-1-$C^{14}$ into the long-chain fatty acids of the ascites cells was relatively poor in the absence, but markedly stimulated in the presence of glucose (Tables I and II). This stimulation is not characteristic of the ascites cells since it has been found also with many solid tumours (Emmelot and Bosch, 1955*b* ; van Vals and Emmelot, 1957).

Incubation of the ascites cells with glucose and acetate-1-$C^{14}$ resulted in a very marked drop in the $C^{14}$-content of the proteins as compared with the concentration of $C^{14}$ found in the proteins after incubation with the radioacetate alone (Tables I and II). The average drop in the $C^{14}$-content of the proteins of the glucose supplemented mammary carcinoma cells was somewhat less than that found with the rhabdomyosarcoma ascites cells, *i.e.* 50 and 72 per cent respectively. This phenomenon has never been observed with any tumour of the solid type (Emmelot and Bosch, 1955*b* ; van Vals and Emmelot, 1957). To see at which concentration of glucose the impairment in the incorporation of $C^{14}$ into the proteins took place, two experiments were carried out with acetate-1-$C^{14}$ and varying amounts of unlabelled glucose (19, 38, 75 and 113 $\mu$mole) using the rhabdomyosarcoma cells. The results are illustrated in Fig. 1. At the lowest concentration of glucose (one-fourth of that used in the experiments of Table I) the $C^{14}$-content of the proteins was already markedly diminished, at the next higher concentration this effect was more marked and then remained constant.

By contrast, the stimulation of fatty acid synthesis was, in both experiments, the most pronounced at the lowest glucose concentrations tested. The specific activities of the carbon dioxide were higher at all glucose concentrations and the total amount of acetate-1-$C^{14}$ oxidized was only in one experiment with the 75 $\mu$mole concentration of glucose somewhat smaller than the values found in the absence of glucose.

It is known that pyruvate does not give rise to the Crabtree effect in ascites tumour cells (Racker, 1956). Unlabelled pyruvate (75 $\mu$mole), added to mammary ascites cells (750 mg.), and incubated with radioacetate for 2·5 hours, caused a

FIG. 1.—Incorporation of $C^{14}$ from acetate-1-$C^{14}$ into carbon dioxide, long-chain fatty acids (FA) and proteins by ascites cells of a rhabdomyosarcoma in the presence of various concentrations unlabelled glucose.

The results of two experiments are illustrated : △—△ with ascites cells containing 10·8 mg. N (Kjeldahl) and ○—○ *ibidem* 11·0 mg. N. Specific activity of $C^{14}O_2$ (solid lines) and proteins measured as counts per minute of an " infinitely " thick layer of $BaCO_3$, or protein on 1·1 cm.$^2$ area.

For convenience of representing the graphs, the total activity in $C^{14}O_2$ (dotted lines) is expressed as specific activity × mg. $BaCO_3$ recovered. Calculation showed that 0·77 (glucose absent), 0·88 (19 $\mu$mole glucose present), 1·05 (38 $\mu$mole glucose present), 0·94 (75 $\mu$mole glucose present) and 0·99 (113 $\mu$mole glucose present) × $10^{-3}$ m-mole acetate were incorporated into the carbon dioxide samples of the first experiment, and respectively 0·80, 0·87, 1·10, 0·75 and 1·08 × $10^{-3}$ m-mole acetate in the second experiment.

five-fold drop in the $C^{14}$-content of the proteins (from 110 to 25 c./m.). A corresponding fall was noted in the specific activity of the respiratory carbon dioxide : 12·4 mg. $BaCO_3$ showing 3047 c./m. at " infinite thickness " was recovered in the absence, and 17·8 mg. $BaCO_3$ showing 600 c./m. in the presence of pyruvate. The drop in the $C^{14}$-content of the proteins following pyruvate supplementation was therefore due to a dilution of the citric acid cycle intermediates which yielded the carbon skeletons of the amino acids incorporated into the proteins.*

The pattern of acetate utilization by the rhabdomyosarcoma cells in the presence of glucose was not likely to be due to the particular origin of this tumour, because the $C^{14}$ incorporation into both the proteins and carbon dioxide of slices from normal thigh musle was always found to be stimulated in the presence of unlabelled glucose. In contrast to the depressive effect of glucose on protein labelling by acetate-1-$C^{14}$ in the ascites cells of the rhabdomyosarcoma, no such effect was observed with the solid form of this tumour (Table III). Similarly, only a very small drop (if any) in the $C^{14}$-content of the proteins of spontaneous or transplanted mammary carcinomas from $C_3H$ mice was noted in the presence of unlabelled glucose.

TABLE III.—*Effect of Unlabelled Glucose on the Incorporation of $C^{14}$ from Acetate-1-$C^{14}$ into the Carbon Dioxide, Long-chain Fatty Acids and Proteins of Slices from the Solid Rhabdomyosarcoma*

| Addition | Carbon dioxide m-mole acetate incorporated $\times 10^3$ | Fatty acids m-mole acetate incorporated $\times 10^5$ | Proteins c./m. |
|---|---|---|---|
| A* | 0·29 | 0·67 | 150 |
| A*+G | 0·23 | 4·02 | 150 |

The depressive effect of glucose on the incorporation of tracer from acetate-1-$C^{14}$ into the proteins was thus characteristic of the ascites form of the present tumours and might in some way be connected with the concomitant shift in the utilization of the pathways of intermediary metabolism.

As follows from the $CO_2$ data collected in Tables I and II, and Fig. 1, the drop in the $C^{14}$-content of the proteins could not have been due to a dilution of the tracer in the citric acid cycle intermediates by unlabelled 2-C fragments derived from glucose.

Two other possibilities were, therefore, tested : the impairment by glucose of (i) the formation of certain $C^{14}$-amino acids (probably those derived from the citric acid cycle intermediates), and (ii) the incorporation of these amino acids into the proteins.

Since the incorporation of DL-glutamate-1-$C^{14}$ and DL-leucine-1-$C^{14}$ into the proteins of the mammary carcinoma ascites cells, was essentially the same whether glucose was present or not† (Table IV), the second of these alternatives could be discarded.

* The radioactivity of the long-chain fatty acids was five times higher (557 c./m.) after incubation in the presence of pyruvate than that found in the absence of pyruvate (117 c./m.).

† Incidentally, these experiments show that in these ascites cells the energy for the incorporation process can be derived as well from fatty acid as from glucose catabolic pathways. The incorporation of the amino acids into the proteins of slices from the sarcomatoid ovarian tumour T $26567_s$, on the other hand, was found to be stimulated in the presence of glucose—more than 2-fold in the case of glutamate and 1·5-fold in the case of leucine.

TABLE IV.—*Effect of Glucose on Protein Labelling by and Oxidation of Acetate-1-$C^{14}$, DL-leucine-1-$C^{14}$, and DL-glutamate-1-$C^{14}$ in Mammary Carcinoma Ascites Cells*

300 mg. packed ascites cells incubated for 2 hours in 1·25 ml. Krebs-Ringer phosphate buffer. G = glucose (37 μmole); A = sodium acetate (7·6 μmole); A* = sodium acetate-1-$C^{14}$ (2 μC/7·6 μmole); Gl* = DL-glutamic acid-1-$C^{14}$ (0·33 μC/0·25 μmole); L* = DL-leucine-1-$C^{14}$ (0·33 μC/0·28 μmole).

| Exp. No. | Additions | Protein c./m. | Carbon dioxide mg. $BaCO_3$ | c./m. |
|---|---|---|---|---|
| 1 | A* | 131 | 6·0 | 3709 |
| | A*, G | 47 | 5·1 | 4138 |
| 1 | L*, A | 805 | 7·2 | 2268 |
| | L*, A, G | 797 | 5·7 | 550 |
| 2 | L*, A | 879 | 7·2 | 2082 |
| | L*, A, G | 875 | 6·5 | 344 |
| 1 | Gl*, A | 26 | 6·7 | 1457 |
| | Gl*, A, G | 26 | 5·7 | 3238 |
| 3 | Gl*, A | 31 | 6·6 | 1589 |
| | Gl*, A, G | 32 | 6·1 | 4652 |

It remained then to be demonstrated that the shift from fatty acid to glucose catabolism in the ascites cells was really accompanied by an impairment in the formation of certain amino acids. To this end the nature of these amino acids had to be ascertained. This problem was approached in two ways. First, it was demonstrated that the glucose-induced fall in the protein labelling by acetate-1-$C^{14}$ was accompanied by a fall of comparable magnitude in the radioactivity of glutamate and aspartate isolated from the protein hydrolysates (Table V). Secondly, it was shown that the fixation of $NaHC^{14}O_3$ into the proteins was also markedly depressed in the presence of unlabelled glucose. Incubation of 300 mg. packed mammary carcinoma ascites cells (obtained from the same animals as used for exp. No. 1 of Table IV) with 32 μC $NaHC^{14}O_3$ (0·45 mg.) yielded proteins containing 100 counts per minute at " infinite " thickness, whereas in the presence of unlabelled glucose (36 μmole) the radioactivity amounted to 25 counts per minute. By carboxylation of pyruvate bicarbonate is fixed into oxaloacetate (→ aspartate) and the oxaloacetate-4-$C^{14}$ thus formed is converted into α-ketoglutarate-1-$C^{14}$ (→ glutamate). The tracer cannot reach glycolytic intermediates (formation of phosphopyruvate) since the $C^{14}$ is set free by the decarboxylation of α-ketoglutarate.

TABLE V.—*Radioactivity of Glutamic and Aspartic Acid Derived from the Proteins of Ascites Tumour Cells Labelled by Acetate-1-$C^{14}$*

Amino acids isolated by ion exchange (Amberlite-IR 4 B), plated on 1·1 cm.$^2$ area and assayed at " infinite " thickness.

| Tumour | Substrate | Protein | Glutamic acid | Aspartic acid |
|---|---|---|---|---|
| Rhabdomyosarcoma | A* | 279 | 1080 | 725 |
| | A*+G | 70 | 291 | 225 |
| Mammary carcinoma | A* | 136 | 423 | 288 |
| | A*+G | 57 | 181 | 99 |

The radioactivity appearing in the respiratory carbon dioxide of the experiments of Table IV was also measured and it was found that in the presence of glucose the oxidation of DL-leucine-1-$C^{14}$ was reduced to 20 per cent of that observed in the absence of glucose. In the breakdown of leucine-1-$C^{14}$, acetyl (-1-$C^{14}$)-coenzyme A is formed and the labelled 2-C fragments are oxidized via the citric acid cycle to $C^{14}O_2$. The latter process is not impaired in the presence of glucose as was shown above. However, in the chain of reactions leading from leucine to acetyl-coenzyme A, the sequence :

$$\text{isovaleryl-coenzyme A} \xrightarrow{(-2H)} \beta\text{-methylcrotonyl-coenzyme A} \xrightarrow{(H_2O)} \beta\text{-hydroxy-valeryl-coenzyme A}$$

is essentially of the type found in the fatty acid oxidation cycle (Coon, Robinson and Bachhawat, 1955). The latter reaction may even be catalyzed by crotonase. Since the fatty acid oxidation cycle is impaired in the presence of glucose, the possibility that the same situation is also responsible for the impairment in the breakdown of leucine at the dehydrogenation step may be envisaged.*

In contrast to the oxidation of leucine, the oxidation of DL-glutamate-1-$C^{14}$ by the mammary carcinoma cells was markedly enhanced in the presence of glucose (200–250 per cent, Table IV).

These experiments were carried out with exceedingly small concentrations of highly labelled amino acids in order not to disturb the pool sizes of the free amino acids, and, consequently, the metabolic equilibria of the type $\alpha$-keto acid $\rightleftharpoons$ $\alpha$-amino acid. The latter reaction involving :

$$\alpha\text{-ketoglutarate} + NH^+{}_4 + DPNH \rightleftharpoons \text{L-glutamate} + DPN^+ \quad . \quad . \quad . \quad (1)$$

and coupled with transamination reactions (L-glutamate + $\alpha$-keto acid $\rightleftharpoons$ L-$\alpha$-amino acid + $\alpha$-ketoglutarate), is of vital importance in amino acid metabolism, since the equilibrium of reaction (1) decides whether amino acids are formed or broken down.

Since glucose appears to shift reaction (1) to the left, it may be concluded that less glutamate is formed from the citric acid cycle intermediate $\alpha$-ketoglutarate in the presence than in the absence of carbohydrate. Consequently transamination reactions, *e.g.* leading to aspartate, are also slowed down. This interpretation may account for the decreased incorporation of $C^{14}$ from acetate-1-$C^{14}$ into the amino acids of the protein brought about by glucose. The latter effect, as pointed out above, is, at least as far as our experiments go, characteristic of the ascites tumour cells ; no such effect has ever been consistently observed in this laboratory with a solid tumour. In accordance herewith stands the finding that the oxidation of glutamate by slices of various solid tumours was depressed or not markedly affected in the presence of glucose, whereas the oxidation of leucine was inhibited but to a far lesser extent than in the case of the ascites cells.

Why does glucose impair the formation of glutamate in the ascites cell ? This question can only be answered in a speculative manner at present. Considering

* This conclusion is valid if the transamination reaction leucine + $\alpha$-ketoglutarate $\longrightarrow$ $\alpha$-keto-isocaproate + L-glutamate is not impaired by a lack of $\alpha$-ketoglutarate (see below). The effect of unlabelled glucose on the pattern of incorporation into the proteins and of the oxidation of DL-lencine-1-$C^{14}$ was similar for the two ascites tumours. However, glucose moderately depressed (30 per cent) the incorporation of DL-glutamate-1-$C^{14}$ into the proteins of the rhabdomyosarcoma cells. The oxidation of glutamate by the latter cells in the presence of glucose was 1·5 fold that observed in the absence of carbohydrate.

(1) as the key-reaction, carbohydrate catabolism may lower the steady-state concentrations of either $\alpha$-ketoglutarate or DPNH as compared with the levels reached during fatty acid catabolism. The protein sparing effect of carbohydrate in normal liver has been explained (Miller, Burke and Haft, 1955) on the basis that an active glycolysis enhances the DPNH/$DPN^+$ ratio and thus counteracts amino acid breakdown. This interpretation is exactly opposite to that given to explain the situation in the ascites cell.

Further experiments are being carried out on these points.

SUMMARY

The oxidation of acetate-1-$C^{14}$ by ascites cells of a rhabdomyosarcoma and a mammary carcinoma is hardly, if at all, depressed in the presence of excess unlabelled glucose.

Since the oxygen consumption is markedly less under the latter condition (up to 50 per cent) it is concluded that the oxidative degradation of endogenous fatty acids to their 2-C fragments, but not the passage of these fragments through the citric acid cycle, is primarily inhibited as a result of the glucose supplementation.

Glucose favoured the incorporation of acetate-1-$C^{14}$ into the long-chain fatty acids, but depressed the incorporation of tracer from acetate-1-$C^{14}$ or $HC^{14}O_3^-$ into the trichloroacetic acid-insoluble proteins.

The incorporation of DL-leucine-1-$C^{14}$ or DL-glutamic acid-1-$C^{14}$ into the proteins was not affected by glucose addition but the oxidation of leucine was inhibited whereas that of glutamate was enhanced. The latter observations may serve to explain the depressive effect of glucose on protein labelling by acetate-1-$C^{14}$ which was found to be characteristic for the ascites form of the present tumours.

Our thanks are due to Miss S. H. Nout for technical assistance and to Dr. G. Klein for sending us the ascites tumours.

### REFERENCES

AISENBERG, A. C., REINAFARJE, B. AND POTTER, V. R.—(1957) *J. biol. Chem.*, **224**, 1099, 1115.

BLOCH-FRANKENTHAL, L. AND WEINHOUSE, S.—(1957) *Proc. Amer. Ass. Cancer Res.*, **2**, 189.

BRIN, M. AND MCKEE, R. W.—(1956) *Cancer Res.*, **16**, 364.

CHANCE, B. AND HESS, B.—(1956) *Ann. N.Y. Acad. Sci.*, **63**, 1008.

COON, M. J., ROBINSON, W. G. AND BACHHAWAT, B. K.—(1955) 'Amino Acid Metabolism, (McElroy and Glass, eds.). Baltimore (the Johns Hopkins Press), p. 431.

EMMELOT, P. AND BOSCH, L.—(1955a) *Brit. J. Cancer*, **9**, 327.—(1955b) *Ibid.*, **9**, 339.

JOHNSON, M. S.—(1941) *Science*, **94**, 200.

KUN, E., TALALAY, P. AND WILLIAMS ASHMAN, H. G.—(1951) *Cancer Res.*, **11**, 855.

LYNEN, F.—(1941) *Ann. Chem. J. Liebigs*, **546**, 120.—(1956) Proc. 3rd Internat. Congress Biochemistry, Brussels 1955 (Acad. Press, N.Y.), p. 294.

MILLER, L. L., BURKE, W. T. AND HAFT, D. E.—(1955) *Fed. Proc.*, **14**, 707.

RACKER, E.—(1956) *Ann. N.Y. Acad. Sci.*, **63**, 1017.

SEELICH, F., WEIGERT, W. AND LETNANSKY, K.—(1956) *Z. Krebsforsch.* **61**, 368.

SLECHTA, L., JAKUBOVIC, A. AND SORM, F.—(1955) Resumées des Communications, 3ième Congrès International de Biochimie, Bruxelles 1955, p. 126 (14–39).

VALS, G. H. VAN, AND EMMELOT, P.—(1957) *Z. Krebsforsch.* **62**, 63.